# Health Tract, No. 213

**Published:** 1865

**Authors:** 


HEALTH TRACT. No. 213.

### PHILOSOPHY OF EATING.

THE young eat for three reasons: 1st. To grow; 2d. To keep warm; 3d. To repair waste. Adults eat for the last two purposes; hence all food contains one of two elements, and some kinds both, called nitrogen and carbon. The nitrogen makes flesh, sometimes called muscle, and is the same as lean meat. Carbon makes the fat, and is that which keeps us warm Sugar, starch, arrowroot, oil, butter, suet, and lard have no nitrogen; there is nothing in them to make flesh out of; all the nutriment they afford is carbon, the material for warmth. Infants and young children would soon die, would get so chilly as to freeze, as it were, unless they had something sweet in their food; hence nature has implanted in them an unappeasable taste for sweet things. The thing the new-born infant needs first and always is warmth. Butter, oils, and starches abound also in the heat-producing elements, but they require strong powers of digestion, are applicable to grown-up persons and to the old; hence as we grow old we like fat meats, oils and butter more.

It is in obedience to these laws that Almighty beneficence and wisdom has imparted a relish for oils and fat meats in winter, because extra heat is needed. Greenlanders, whose country is always covered with ice and snow, consider butter and lard and tallow candles and the rankest oils the greatest luxuries conceivable. But rice, on which many of the inhabitants of warm countries chiefly live, is said to contain scarce one per cent of the fat or heat-producing element, while oils have ninety-six per cent of it.

All know how buckwheat cakes are relished in winter; but as spring comes on, we begin to lose our appetite for them. The cakes themselves contain fifty-four per cent of the fat or heat-producing element, and they are made more palatable by spreading butter on them, and adding to this molasses, each being almost entirely (ninety-six per cent,) heat-producing.

But out-door workers eat meat and bread the year round, and never weary of it, because twenty-two per cent of them are flesh-forming, give that much power and strength to work.

